# Recent Advances in Our Understanding of the Link between the Intestinal Microbiota and Systemic Lupus Erythematosus

**DOI:** 10.3390/ijms20194871

**Published:** 2019-09-30

**Authors:** Ji-Won Kim, Seung-Ki Kwok, Jung-Yoon Choe, Sung-Hwan Park

**Affiliations:** 1 Division of Rheumatology, Department of Internal Medicine, Catholic University of Daegu School of Medicine, Daegu 42472, Korea; kimjw689@cu.ac.kr (J.-W.K.); jychoe@cu.ac.kr (J.-Y.C.); 2 Division of Rheumatology, Department of Internal Medicine, Seoul St. Mary’s Hospital, College of Medicine, The Catholic University of Korea, Seoul 06591, Korea; seungki73@catholic.ac.kr

**Keywords:** systemic lupus erythematosus, microbiota, host microbial interactions, molecular mimicry, autoantibodies, interferon type I

## Abstract

Systemic lupus erythematosus (SLE) is an autoimmune disease featuring enhanced expression of type I interferon (IFN) and autoantibody production triggering inflammation of, and damage to, multiple organs. Continuing research efforts focus on how gut microbes trigger systemic autoimmunity and SLE. The gut microbial communities of mice and humans with lupus have been investigated via high-throughput sequencing. The Firmicutes-to-Bacteroidetes ratio is consistently reduced in SLE patients, regardless of ethnicity. The relative abundance of *Lactobacillus* differs from the animal model used (MRL/lpr mice or NZB/W F1 mice). This may indicate that interactions between gut microbes and the host, rather than the enrichment of certain gut microbes, are especially significant in terms of SLE development. *Enterococcus gallinarum* and *Lactobacillus reuteri*, both of which are possible gut pathobionts, become translocated into systemic tissue if the gut epithelial barrier is impaired. The microbes then interact with the host immune systems, activating the type I IFN pathway and inducing autoantibody production. In addition, molecular mimicry may critically link the gut microbiome to SLE. Gut commensals of SLE patients share protein epitopes with the Ro60 autoantigen. *Ruminococcus gnavus* strain cross-reacted with native DNA, triggering an anti-double-stranded DNA antibody response. Expansion of *R. gnavus* in SLE patients paralleled an increase in disease activity and lupus nephritis. Such insights into the link between the gut microbiota and SLE enhance our understanding of SLE pathogenesis and will identify biomarkers predicting active disease.

## 1. Introduction

Systemic lupus erythematosus (SLE) is a prototypic autoimmune disease affecting multiple organs, particularly in females of childbearing age. SLE is characterized by autoantibody production and immune complex deposition reflecting dysregulation of both the innate and adaptive immune systems. Loss of tolerance to various self-antigens is a critical feature of SLE. Nuclear antigens released during apoptosis are normally cleared without activating the immune system. However, in SLE, external stimuli such as ultraviolet (UV) light, an infection, or a toxin may increase the apoptotic cell load [1]. Increased levels of nucleic acid-containing cell debris activate the type I interferon (IFN) pathway via the action of nucleic acid recognition receptors such as the Toll-like receptor (TLR) 9 or TLR7 [2,3]. Type I IFNs and other cytokines promote B cell maturation and survival [1,4,5]. B cell hyperactivity is a hallmark of SLE; the cells produce high-affinity autoantibodies against self-antigens and, thus, damage tissues. Autoantibodies and immune complexes mediate inflammation and tissue damage by activating complement and binding to Fc gamma receptors on inflammatory cells [1]. Lupus autoantibodies are high-affinity and produced by B cells, which have undergone class-switch recombination; it suggests that T cells are involved in B cell activation and autoantibody production. Activated T cells release proinflammatory cytokines and activate B cells to secrete autoantibodies. An increase in follicular helper T cells and defects in regulatory T (Treg) cells have been implicated in SLE pathogenesis [6,7]. SLE patients also exhibit an expansion of double-negative (CD4^-^CD8^-^) T cells, which can provide help for pathogenic autoantibody production [8,9]. Thus, activation of both B cells and T cells enhances innate and adaptive immune responses toward autoimmunity [10,11].

The etiology of SLE is multifactorial. Environmental and genetic factors interact to trigger SLE development. Differences in SLE development between monozygotic twins suggest that environmental triggers are important in terms of lupus development in genetically susceptible individuals [12]. UV light, hormones, infections (e.g., Ebstein-Barr virus and cytomegalovirus), cigarette smoking, toxins (e.g., silica dust), and drugs are all potential triggers of SLE [13,14]. UV light induces DNA damage and keratinocyte apoptosis, increasing exposure to autoantigens [15]. UV light can induce T cell activation in lupus-prone mice via type I IFN-dependent suppression of Treg cells [16]. Estrogen and prolactin activate the immune system and are associated with active SLE [17,18]. Indeed, oral contraceptive use and postmenopausal hormone therapy increase the risk of SLE [19,20].

Recently, growing evidence suggests that the microbiota plays a role in SLE [21,22]. The gut microbiota of SLE patients is associated with an imbalance in the proportions of T helper 17 (Th17) and Treg cells [23]. In addition, the development of antinuclear antibodies is affected by the composition of the early gut microbiota [24]. Advances in metagenomic sequencing techniques (16S rRNA and whole-genome sequencing) have enabled the study of the mechanistic role played by the gut microbiota in many diseases. In this review, work on the gut microbiota of mice and humans with SLE will be summarized, and our current understanding of the link between the gut microbiota and SLE will be explained.

## 2. The Role of the Gut Microbiota in Human Health and Disease

Humans host diverse microorganisms in the gut lumen. The number of microorganisms varies along the gut, being 10^11^ cells/g in the ascending colon, 10^7–8^ cells/g in the distal ileum, and 10^2–3^ cells/g in the proximal ileum and jejunum; over 2000 species of bacteria have been described [25]. Metagenomic sequencing has revealed that gut microbes encode 150-fold more genes than the human genome [26]. It is now realized that the microbiota does not just reside in the gut; the bacteria interact with the human host to maintain gut homeostasis. By metabolizing dietary carbohydrates and proteins, the gut microbiota provides nutrients and energy to both the intestinal epithelial cells and peripheral tissues [27]. The gut microbiota also releases several factors affecting human metabolism [28]. The gut microbiota maintains the gut barrier function and the anaerobic environment of the gut [29]. Importantly, the gut microbiota shapes the physiological immune responses of the intestine [30,31]. The host immune system protects against harmful gut pathogens but tolerates gut commensals [31]. This host immunity may be possible due to pre-immune immunoglobulin repertoires shaped by the symbiotic gut microbes [32]. However, dietary changes and the use of antibiotics can affect the gut microbiota and disrupt gut homeostasis [33,34]. When the alteration of gut microbial composition and function (this is termed gut dysbiosis) appears, aberrant immune responses develop, both locally and systemically.

Several diseases are associated with gut dysbiosis. Early studies found that obesity correlated with a shift in the gut microbial community [35,36]. Transmission of “obese microbiota” to germ-free mice increased body fat levels [36]. Immune-mediated inflammatory diseases such as inflammatory bowel disease (IBD) [37,38], type I diabetes [39,40], allergies and asthma [41,42,43], rheumatoid arthritis (RA) [44,45,46,47,48], and multiple sclerosis [49,50] and cancers such as colorectal, gastric, and liver cancer [51] are also associated with alterations in the gut microbial community. Thus, the gut microbiota impacts the host immune system during disease establishment; modulation of the gut microbiota might, therefore, be of therapeutic utility.

## 3. The Gut Microbiome in Murine Lupus

Spontaneous and induced animal models of lupus have been employed to determine the effect of the gut microbiota on lupus. MRL/lpr mice with the *lpr* mutation in the gene encoding Fas protein can spontaneously develop many lupus autoantibodies and lupus manifestations [52]. The gut microbiota of MRL/lpr lupus-prone mice exhibit a low proportion of Lactobacillaceae and are enriched in Lachnospiraceae [53]. Lactobacillaceae includes bacteria that produce lactic acid as an end-product of carbohydrate metabolism. *Lactobacillus* constitutes the microbiota of human gastrointestinal and genitourinary tracts. Some *Lactobacillus* species are used as probiotics since they are known to have anti-inflammatory properties [54]. Lachnospiraceae (belongs to Clostridia order) are major components of the human gut microflora and includes many butyrate-producing bacteria [55]. Lachonospiraceae protects against colon cancer and may influence obesity; both are mediated by butyrate production [55]. The severity of lupus disease indices (lymphadenopathy and glomerulonephritis) was inversely correlated with the relative abundance of Lactobacillaceae and positively correlated with the relative abundance of Lachnospiraceae [53]. The relative lack of *Lactobacillus* spp. was most prominent before (not after) disease onset [56]. Thus, *Lactobacillus* might play a preventive role in terms of lupus pathogenesis. Indeed, *Lactobacillus* addition reduced proteinuria and levels of lupus autoantibodies and improved the renal pathology scores in/of MRL/lpr mice [56].

However, *Lactobacillus* played an opposite role in studies using different lupus mouse models. NZB/W F1 mice, another spontaneous lupus model, developed lupus slowly [57]. Luo et al. investigated the dynamics of the gut microbiota of NZB/W F1 mice [58]. The gut microbiota changed throughout the mouse lifetime; the changes were particularly marked before and after lupus onset [58]. The relative abundance of *Lactobacillus* increased dramatically during lupus development; this rise was reversed by dexamethasone [58]. The relative abundance of *Lactobacillus* species tended to correlate positively with poorer renal function and higher-level systemic autoimmunity [58].

In TLR7-dependent mouse models, the lupus-prone TLR7.1 Tg mice and imiquimod (a TLR7 agonist)-induced mice, *Lactobacillus reuteri* alone exacerbated lupus [59]. In fecal samples of such lupus-prone mice, *L. reuteri,* the genus Desulfovibrio, and the family Rikennellaceae were enriched. However, only *Lactobacillus* spp. (*L. reuteri* and *L. johnsonni*) were translocated to internal organs, and only *L. reuteri* (thus not *L. johnsonni*) induced the IFN gene signature and systemic autoimmunity. Resistant starch (fermented to short-chain fatty acids) suppressed *L. reuteri* growth both in vitro and in vivo. This reduced gut epithelial permeability in vivo, and decreased type I IFN expression and improved lupus nephritis.

The effects of relative abundance of *Lactobacillus* and the changes mediated by *Lactobacillus* differ among the various mouse models of lupus. The mechanisms underlying lupus development are not identical among the mouse strains; the gut microbiota compositions and associated host interactions vary. IFN-α plays the predominant role in TLR7-dependent mouse models, whereas IFN-γ is more important in MRL/lpr mice [60]. In addition, the genus Lactobacillus includes many species that may play different roles in lupus pathogenesis. Similar observations have been made in models of other autoimmune diseases, such as RA; inoculation with *Prevotella copri* triggered arthritis, whereas inoculation with *Prevotella histicola* suppressed arthritis [61]. In addition, unsequenced species may exist from the 16S rRNA sequencing method, as shown in previous animal studies [58]. Work on the gut microbiome in murine lupus models is summarized in Table 1. Below, whether similar results have been obtained in humans is explored.

## 4. The Gut Microbiome in Human Lupus

The human gut microbiome consists principally of the phyla Firmicutes and Bacteroidetes [64,65]. The ratio of Firmicutes to Bacteroidetes has been investigated in patients with various diseases and has been shown to increase with obesity [66]. In SLE patients, on the other hand, the relative abundance of Firmicutes is decreased, and that of *Bacteroidetes* is increased, showing the decreased ratio of Firmicutes to Bacteroidetes compared to healthy controls [67,68,69,70]. Most studies were performed in female SLE patients; the results in Caucasians and Asians were consistent. One study found no significant difference in the Firmicutes/Bacteroidetes ratio between SLE patients and healthy controls (the study included both male and female patients; the SLEDAI ranged from 0 to 13) [58]. Rather, the phylum Proteobacteria was enriched in SLE patients.

The relative abundance of *Lactobacillus* spp. was increased in the gut microbiota of SLE patients compared to healthy controls, consistent with the rise in the relative abundance of *L. reuteri* in lupus-prone mice [59]. However, the *Lactobacillus* spp. spectrum was not clarified in human fecal samples.

Azzouz et al. were the first to describe the overabundance of *Ruminococcus gnavus* (of the family Lachnospiraceae) in the gut microbiota of 61 female SLE patients varying in terms of disease activity [71]. Gut expansions of *R. gnavus* reflected the extent of disease activity and were prominent in patients with lupus nephritis [71]. Interestingly, strain RG2, but not strain RG1, cross-reacted with anti-double-stranded (dsDNA) antibodies [71]. Patients with active lupus nephritis exhibited an immune response to RG2; this finding was validated in two small independent cohorts (that included male patients and of diverse ethnicity) [71]. Thus, the level of serum anti-RG2 IgG may serve as a surrogate marker of SLE disease activity and lupus nephritis. However, it remains unclear whether an elevated serum level of anti-RG2 IgG response is specific for SLE. *R. gnavus* was also abundant in fecal samples from spondyloarthritis patients; the levels reflected disease activity [72]. Moreover, transient increases in *R. gnavus* levels have been observed in the gut microbiome of patients with IBD; the *R. gnavus* strains of IBD patients and healthy controls differed [73]. Works on the gut microbiome of human lupus are summarized in Table 2.

## 5. Potential Mechanisms Linking the Gut Microbiota to SLE

### 5.1. The Leaky Gut and Gut Microbiota Translocation

Intestinal epithelial integrity is maintained in healthy subjects. Normally, gut-resident bacteria do not transfer to internal organs. However, if the gut barrier is disrupted, gut commensals are exposed to the host immune systems of various organs. A “leaky gut” initiates systemic autoimmunity. The role of an impaired gut barrier in lupus pathogenesis has been well-studied in mice [56,59,63]. The levels of intestinal epithelial tight junction proteins are decreased in lupus-prone MRL/lpr and (NZW × BXSB) F1 mice [56,63]. Given the leaky gut, bacteria are translocated to systemic tissues to activate antigen-presenting cells that, in turn, migrate to mesenteric lymph nodes (MLNs) [56]. Next, CD4^+^ T cells are activated, and inflammatory cytokines such as IL-6 are released. IL-6 plays a pivotal role in lupus progression in MRL/lpr mice by inducing autoantibody production by B cells and suppressing the activities of Treg cells [74,75].

Antibiotic-treated or germ-free lupus-prone mice exhibit enhanced gut integrity and lower-level systemic autoimmunity [59,63]. Thus, the gut microbiota affects intestinal integrity and lupus development. The next question is: “Which gut pathobiont(s) play a direct role(s) in the gut microbiome-host interaction?” Manfredo-Vieira et al. were the first to define the role played by *Enterococcus gallinarum* in gut leakage and bacterial translocation inducing the development of lupus autoantibodies [63]. *E. gallinarum,* a gut commensal, was detected in the mesenteric veins, MLNs, and liver of (NZW × BXSB) F1 mice both via tissue cultures and in situ assays; C57BL/6 mice did not exhibit systemic bacterial growth [63]. *E. gallinarum* increased the frequencies of plasmacytoid dendritic cells (DCs), the source of the SLE IFN signature [63]. In addition, *E. gallinarum*-induced autoantigens stimulated the aryl hydrocarbon receptor (AhR)-CYP1A1 pathway, in turn triggering Th17 cell activation and anti-dsDNA antibody production [63]. Lupus autoantibody production was induced after colonization by *E. gallinarum* alone, but not after colonization by *Enterococcus faecalis* or *Bacteroides thetaiotaomicron* [63]. Immunization of lupus-prone mice against *E. gallinarum* limited bacterial translocation and the lupus manifestations. Thus, *E. gallinarum* per se may be a gut pathobiont in the SLE context. Notably, *E. gallinarum* has been found in liver biopsy samples of both SLE patients and autoimmune hepatitis patients [63]. Human SLE studies have revealed gut barrier impairments. The luminal contents not only leaks into systemic tissues but also systemic Ig leaks into the gut lumen [63,71].

Although *E. gallinarum* was present in the small intestine, the bacterium was not detected in the feces of lupus-prone mice via 16S rRNA sequencing [63]. This may indicate that the overabundance of gut pathobionts is not required for the bacterial translocation. Rather, the contributions of gut pathobionts to the development of leaky gut and activation of immune responses may be more important in terms of lupus pathogenesis.

### 5.2. Molecular Mimicry

Autoantibodies to the 60 kDa Ro protein are common in SLE patients. It was found early that the lupus autoantigen Ro60 cross-reacted with the Ebstein-Barr virus nuclear antigen-1 (EBNA-1), suggesting that lupus humoral autoimmunity was initiated via molecular mimicry of EBNA-1 and Ro60 antigens [76].

Recently, commensal bacteria have been shown to trigger autoreactive T and B cell responses in susceptible individuals. Peptides derived from the oral, gut, skin, and vaginal microbiotas activated Ro60-reactive T cells [77]. Greiling et al. found that bacteria expressed orthologs of the human Ro60 autoantigen [69]. Ro60 ortholog-containing commensals were common in the oral, skin, and gut environments of humans, including SLE patients, and delivered antigens to immune cells [69]. Commensal-reactive T cell clones from SLE patients cross-reacted with the human Ro60 autoantigen, and human Ro60-reactive T cell clones cross-reacted with the commensal Ro60 orthologs [69]. In addition, sera from anti-Ro60-positive-patients with SLE immunoprecipitated bacterial ribonucleoprotein complexes containing Ro60 orthologs [69]. Furthermore, C57BL/6 mice colonized with (only) the ortholog Ro60-containing gut commensal *Bacteroides thetaiotaomicron*, which expressed human anti-Ro60 antibodies in the blood [69]. In summary, both antibody and T cell cross-reactivity trigger human anti-Ro responses in SLE patients.

Intestinal expansion of *R. gnavus* correlated with lupus disease activity and lupus nephritis [71]. *R. gnavus* overgrowth was associated with the development of serum antibodies against strain RG2. Patients with high titers of IgG anti-RG2 antibodies exhibited active lupus nephritis. Of the various strains of *R. gnavus*, extracts of RG2, but not RG1, cross-reacted with lupus anti-dsDNA antibodies, suggesting that cross-reactivity was strain-restricted. Cell wall lipoglycans of the RG2 strain contained the antigenic properties for anti-dsDNA antibody responses. Molecular mimicry between RG cell wall moieties and native DNA molecules may trigger or exacerbate SLE and lupus nephritis.

### 5.3. Sexual Dimorphism of the Gut Microbiota

SLE is more common in females than in males. Both female and male lupus-prone mice spontaneously develop lupus. However, female mice develop a more severe disease than male mice. A previous study from Zhang et al. investigated the gut microbiota of female and male MRL/lpr mice; the patterns differed markedly [53]. Female MRL/lpr mice contained more Lachnospiraceae and less Bifidobacterium than male MRL/lpr mice, but the levels of Lactobacillaceae were comparable. Female MRL/lpr mice had more Lachnospiraceae than female MRL control mice, but male MRL/lpr mice and MRL control mice did not differ significantly. Thus, the more severe disease of female (compared to male) lupus-prone mice may result from the difference in gut microbial communities, such as the greater abundance of Lachnospiraceae [53].

The sex difference in the gut microbiota emerges in adulthood, suggesting that the gut microbiota is affected by sex hormones [78]. Castration of male mice reversed the difference in the gut microbiota between male and female mice, demonstrating the gut microbiota change was androgen-dependent [79]. Testosterone protects against autoimmune diseases such as type I diabetes, associated with the sex-specific gut microbiome profile [78]. Non-obese diabetic (NOD) mouse, a mouse model of type I diabetes, shows a strong female bias in type I diabetes incidence [79]. Transfer of gut microbiota from male NOD mice to female NOD mice altered the microbiome composition of the recipients, resulting in increased testosterone levels, suppressed pancreas islet inflammation, and autoantibody production, and reduced diabetes incidence in female mice [78]. Yurkovetskiy et al. found that only certain gut microbiota lineages, not all the expanded gut microbiota lineages, in male mice could induce the protection from diabetes, and testosterone, a second signal, was required for this protection [79]. Thus, sex hormones and the gut microbes interact to protect male NOD mice from diabetes (this is termed as a “two-signal model”) [79]. Enhanced IFN-γ signaling was suggested to be one of the protective pathways involved in the microbes-hormones axis supporting sex bias of NOD mice [79]. Although exact mechanisms are not fully investigated, sex hormones and the gut microbiota may participate together in the sexual dimorphism of lupus observed in mice and humans.

Sex hormones may influence the effects of the gut microbiota in lupus-prone mice. *Lactobacillus* treatment reversed proteinuria and improved the renal pathology scores in female MRL/lpr mice [56]. In male MRL/lpr mice given *Lactobacillus*, both mock-castrated and castrated mice exhibited increased gut colonization of *Lactobacillales*. However, only castrated mice evidenced reduced proteinuria and improved renal pathology scores; mock-castrated mice did not. In summary, *Lactobacillus* was therapeutic in both female lupus-prone mice and castrated male lupus-prone mice, but not in intact male mice. Thus, androgenic hormones may suppress the therapeutic effect of the gut microbiota.

**Figure 1:**
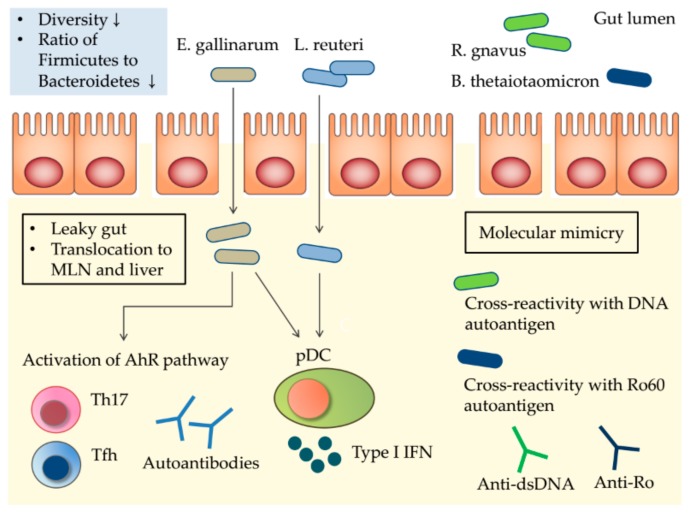
Potential mechanisms by which the gut microbiota triggers the autoimmunity of systemic lupus erythematosus (SLE). SLE patients exhibit restricted gut microbial diversity, with the expansion of possible pathobionts. Impaired gut permeability (a “leaky gut”) allows translocation of pathobionts to mesenteric lymph nodes (MLNs) and the liver. *E. gallinarum* delivers ligands to the aryl hydrocarbon receptor (AhR), and activation of the AhR pathway induces proliferation of Th17 and Tfh cells; systemic autoantibody production follows. *E. gallinarum* induces type I interferon (IFN) expression by plasmacytoid dendritic cells (pDCs) and hepatocytes. Toll-like receptor 7 (TLR7)-dependent translocation of *L. reuteri* increases pDCs numbers and type I IFN expression, exacerbating SLE. Molecular mimicry of human autoantigens by bacterial orthologs triggers cross-reactive T and B cell responses, inducing autoimmunity. ↓, decreased.

Potential mechanisms of the gut microbiota triggering the autoimmunity of SLE are summarized in Figure 1.

## 6. Conclusions

Exploration of the gut microbiome in murine and human lupus has afforded new insights into the role played by the microbiota in SLE. Several taxa are either enriched or depleted in the lupus gut, as revealed via high-throughput sequencing. The ratio of Firmicutes to Bacteroidetes is reduced in SLE patients, regardless of ethnicity. Most studies were performed in female mice or patients since sex hormones can affect the gut microbiota. Gut pathobionts interact with hosts by translocating to systemic tissues and, thereby, activating the immune system. *E. gallinarum* and *L. reuteri* cross the gut epithelium to induce type I IFN expression and anti-dsDNA antibody production. Molecular mimicry is critical in terms of autoimmunity induction by gut microbes. Gut commensals from humans (including SLE patients) contain proteins with epitopes homologous to those of the Ro60 autoantigen. Such proteins bind to B and T cells to activate (inappropriate) immune responses. In addition, overexpression of specific gut microbes indicates increased disease activity and predicts lupus nephritis. The gut microbiome aids our understanding of SLE pathogenesis and can serve as a biomarker predicting active disease.

## Figures and Tables

**Table 1:** Works investigating the gut microbiome in murine lupus.

Study	Mice	Results
Zhang et al. 2014 [53]	MRL/lpr mice	Lactobacillaceae ↓, Lachnospiraceae ↑, Ruminococcaceae ↑, Rikenellaceae (genus Alistipes) ↑ in lupus-prone mice compared to control mice. Gut microbiota of lupus-prone mice distinct between the sexes. Higher abundance of Lachnospiraceae and lower abundance of Lactobacillaceae associated with the lupus disease indices of lymphadenopathy and glomerulonephritis.
Mu et al. 2017 [56]	MRL/lpr mice	Lactobacillales ↓ and a leaky gut evident in lupus-prone mice. Lactobacillus treatment enhanced gut mucosal barrier, suppressed gut inflammation, and attenuated lupus nephritis.
Luo et al. 2018 [58]	NZB/W F1 mice	Gut microbiota changed before and after disease onset in lupus-prone mice. Species in the genera Clostridium, Dehalobacterium, Lactobacillus, Oscillospira, Dorea, Bilophila, AF12 and an unnamed genus within the family Ruminococcaceae increased during lupus progression. The relative abundance of *Lactobacillus* tended to be associated with poorer renal function and more extensive systemic autoimmunity.
Johnson et al. 2015 [62]	SNF1 mice	Abundant Rikenellaceae associated with more rapid lupus progression. Giving mice acidic water delayed lupus development compared to intake of neutral water.
Manfredo Vieira et al. 2018 [63]	(NZW × BXSB) F1 mice	*Enterococcus gallinarum* detected in feces, the small intestine, and the liver via culture-based PCR. Translocation of the gut commensal *E. gallinarum* to the liver triggered type I interferon expression and anti-dsDNA antibody production.
Zegarra-Ruiz et al. 2019 [59]	TLR7-dependent spontaneous and induced mice	Enrichment of fecal *Lactobacillus reuteri* and translocation of *L. reuteri* in lupus-prone mice. *L. reuteri* exacerbated lupus by inducing the pDC activation, type I interferon expression, and enhancing glomerulonephritis.

↑, higher abundance or enriched; ↓, lower abundance or depleted.

**Table 2:** Works investigating the gut microbiome in human lupus.

Study	Patients [F:M]	Results
Hevia et al. 2014 [67]	20 SLE patients [20:0]	Bacteriodetes ↑ and Firmicutes/Bacteroidetes ratio ↓ in SLE patients compared to healthy controls.
He et al. 2016 [68]	45 SLE patients [45:0]	Firmicutes ↓, Bacteriodetes ↑, and Firmicutes/Bacteroidetes ratio ↓ in SLE patients compared to healthy controls. Rhodococcus, Eggerthella, Klebsiella, Prevotella, Eubacterium, Flavonifractor, and incertae sedis enriched and Dialister and Pseudobutyrivibrio depleted in SLE patients.
Luo et al. 2018 [58]	14 SLE patients [10:4]	Firmicutes/Bacteroidetes ratio not significantly different between SLE patients and healthy controls. Proteobacteria ↑, Odoribacter ↓, and Blautia ↑ in SLE patients.
Greiling et al. 2018 [69]	16 SLE and 2 SCLE patients [17:1]	Firmicutes/Bacteroidetes ratio ↓ in SLE patients compared to healthy controls. Commensal bacteria containing Ro60 orthologs common in humans, including SLE patients.
van der Meulen et al. 2019 [70]	30 SLE patients [28:2]	Firmicutes/Bacteroidetes ratio ↓, Bacteroidetes ↑, Bacteroides ↑, Alistipes ↑, Proteobacteria ↑ in SLE patients compared to healthy controls.
Zegarra-Ruiz et al. 2019 [59]	28 SLE patients	*Lactobacillus* spp. enriched in SLE patients compared to healthy controls.
Azzouz et al. 2019 [71]	61 SLE patients [61:0]	The abundance of *Ruminococcus gnavus* associated with lupus disease activity and lupus nephritis.

F, female; M, male; SCLE, subacute cutaneous lupus erythematosus, ↑, higher abundance or enriched; ↓, lower abundance, depleted, or decreased.

## Abbreviations

DNA	Deoxyribonucleic acid
rRNA	Ribosomal ribonucleic acid
SLEDAI	Systemic lupus erythematosus disease activity index
